# Supplementary material for: A qualitative exploration of clinicians’ strategies to communicate risks to patients in the complex reality of clinical practice
Source: PLoS One. 2020 Aug 13;15(8):e0236751. doi: 10.1371/journal.pone.0236751 (PMC7425874; doi:10.1371/journal.pone.0236751)
Supplement: S1 Appendix — (PDF) [file pone.0236751.s001.pdf]

S1 Appendix: Semi-structured Interview Guide

1. Introduction of qualitative Interview and Verbal consent

Length: 30-45 minutes

Introduction of interviewee.

Primary goal: The improvement of risk communication in the context of clinical conversations in daily clinical practice in young doctors (General Practitioners or specialists in training).

Therefore, your experiences and opinions regarding verbal and nonverbal communication examples will be extracted. We search for **meaningful examples** that can be used to communicate risks to patients in daily practice (without a particular tool such as decision aid at hand). Those could comprise **practical language (narratives) of verbal analogies or metaphors. Illustrations of numerical risk communication strategies and visualizations like icon arrays or other graphical illustrations including self-made drawings.**

The examples should not only be based on potential effectiveness, but also on feasibility and acceptability for use in daily practice.

Formalities:

1. **Intended use for interview data:** The interview data will be used for analysis within the ZonMW project and the thesis of Romy Richter.
2. **Permission for tape recording and note-taking:** With your permission, I will audiotape and take notes during the interview. The recording is to accurately record the information you provide and will be used for transcription purposes only. If you choose not to be audiotaped, I will take notes instead. If you agree to being audiotaped but feel uncomfortable or change your mind for any reason during the interview, I can turn off the recorder at your request. Or if you don't wish to continue, you can stop the interview at any time.
3. **Means to protect confidentiality and anonymity**: Your study data will be handled as confidentially as possible. Therefore, I will not mention your name during the interview, but approach you in an anonymized manner by using 'Doctor K'. If results of this study are published or presented, no personally identifiable information will be used. I will transcribe the audio recordings as soon as possible after the interview, and then destroy the tapes. When the research is completed, I will save the transcriptions and other study data for possible use in future research done by myself or others. I will retain these records for up to 10 years after the study is over.
4. **Means to guarantee trustworthiness**: The collected data, the analysis and the conclusion is going to be shared with you. For a member check, I will send the

verbatim typed interview to you so that you could correct and clarify provided information, if necessary.

Obtaining verbal consent from the interviewee: Would you like to participate in this interview?

- Do you have any question before I start the interview?

START THE AUDIOTAPE

Thank you for your consent to be interviewed, after I have informed you on the goal of this work.

In order to protect your privacy, I will not name you by your name in the following interview. Instead I will utilize the abbreviation Dr. K to approach you.

2. Personal and practice characteristics

Main question:

- Before we discuss how you communicate risks in daily practice, I would like to hear more about you. Could you tell me a bit about your professional role?
    - Role in project network, job title, responsibilities

- Why you are motivated to discuss this topic?

Prompt questions:

- How many years of working experience do you have as a clinician?
- What is your attitude towards SDM?

3. Risk communication definition

- **Please explain in two sentences:** What is your definition of risk communication in the context of clinical conversations within the concept of shared decision making?

4. Risk communication illustration from daily practice

I would like to know about **concrete RC manners/routines** that you have **used in real practice** with your patient. In the following, I would like you to reflect on **what trainees could use to communicate risks to patients without a particular tool such as decision aid at hand.**

1. First of all, I would like to ask **if you communicate risk in daily practice**?

**If YES:**	**If NO:**
1. Do you remember an example of a patient case when you communicated risk?	1. What would be your idea on how to communicate risk in daily clinical practice without a DA at hand? Could you please give me a concrete example?
2. Can you take me into this clinical consultation and give me some practical examples about your way to communicate risk?	**Examples:** Examples could comprise visual analogies such as narratives, language, metaphors or visualizations like icon arrays or other graphical illustrations.
**Prompting question:**<br>• What risk did you communicate? Disease; Method<br>• How did you communicate risk? Language example	**Prompting question:**<br>• What risk would you communicate? Disease; Method<br>• How would you communicate the risk? Language example

Prompting questions for lack of concrete examples:

I have prepared a patient case that you could use as a teaser to illustrate your manner of communicating risk:

1. Imagine I would be Mrs. Jones/Jane/Mr. Davies how would you phrase the risk of mammography screening/getting a heart attack? Imagine I would be Mrs. Jones/Jane/Mr. Davies how would you phrase the risk of her/him having breast cancer/a heart attack?
   a. Would you use a visual format such as a pictogram?
   b. Would you use verbal analogies such as a metaphor?

Commented [RR1]: After the first interviews it became obvious that the patient cases, were not necessary to make the participants talk about their risk communication experiences. Moreover, most interviewees spontaneous reflected on their own cases with regard to this topic. However, the patient cases were left in the interview guide in case of unexpected need but were not actively integrated in the interview

Mrs. Jones dilemma mammography screening

Mrs. Jones has just celebrated her 45th birthday. She is fit and well and takes no regular drugs. She comes to the surgery to discuss mammography screening. Mrs. Jones has no family history of cancer, had her first period aged 14 years and her first child aged 26. Her sister has told her that a mammogram will detect a cancer before she feels a lump, so that any cancer will be diagnosed earlier, which "can only be a good thing". Mrs. Jones is more skeptical, having read stories in the press of women who had mammograms and biopsies and were then told it was a "false alarm". She wishes to know more about the benefits and harms of mammography screening before making a final decision.

Jane: mammography screening and abnormal finding

Jane is a woman in her 40s who is at average risk of developing breast cancer sometime during her life. She decides to have a mammogram to screen for breast cancer. She gets a call from her doctor saying that the result of the mammogram was abnormal and that she needs to have more tests to determine if she has breast cancer.

Mr. Davies- CVD/Lifestyle change

Mr. Davies has just celebrated his 56th birthday. He is currently feeling well but wanted to have a check-up by his GP as his father died of a heart attack when he was 49. Mr. Davies smokes 30 cigarettes a day. He drinks around 20 units of alcohol a week and takes very little exercise. He works at a local supermarket.
Examination reveals a body mass index of 32 kg/m2 and blood pressure of 140/83 mmHg. Blood test record a total cholesterol level of 6.8 mmol/litre with a high-density lipoprotein cholesterol of 0.9 mmol/litre.

Total cholesterol/HDL=7.5 → according to the Dutch Guidelines for GP´s this results in a 10-year risk of 24% and an indication for recommending preventive drugs (lipid lowering or blood pressure lowering drugs)

Commented [RR2]: After the first interview we adjusted some numbers in the CVD patient case

5. *Risk communication methods*

So far you mentioned … as strategies to communicate risks. In general, with which other risk communication manners/routines are you familiar to use in daily practice?

- How do you deal with (depending on what interviewee already reported)
  - Framing
  - Numerical RC: Natural Frequency, Percentage, RR(R)/AR(R)
  - Verbal RC: Verbal anologies or metaphors
  - Visual RC: Self-made drawings or existing visual tools
  - Uncertainty: aleatoric and epistemic uncertainty

Commented [RR3]: After the first two interviews the interviewee used the following structure concerning the RC methods: After letting the interview freely talk about his risk communication strategies, the interviewee paraphrased the content and if a certain risk communication category (see figure 1) was missing the interviewer asked explicitly for it. However, phrasing of questions were broad enough to leave the interviewee latitude to answer.

Commented [RR4]: After the first two interviews the terms aleatoric and epistemic uncertainty were actively distinct and used when asking about uncertainty

6. *Acceptability and feasibility of RC methods for GP trainees in daily work*

Thank you for your rich ideas.

Based on your experiences and expertise

1. Which manner of communicating risks to patients do you like best?
2. Which way of risk communication would you recommend to others for utilization in daily clinical practice?

7. *Ending of interview*

Thank you for your participation. I really appreciate that you took time for this interview.

I have no further questions. Is there anything else that you would like to add before we finish the interview?

STOP AUDIOTAPE

After end of the Interview: What did you thought about the interview? Do you have any ideas for improvement?
