# Supplementary material for: A qualitative exploration of clinicians’ strategies to communicate risks to patients in the complex reality of clinical practice
Source: PLoS One. 2020 Aug 13;15(8):e0236751. doi: 10.1371/journal.pone.0236751 (PMC7425874; doi:10.1371/journal.pone.0236751)
Supplement: S2 Appendix — (PDF) [file pone.0236751.s002.pdf]

S2: Patient Cases

Table of Contents

S2: Patient Cases ... 1

Risk estimation together with patient for rare disease/infection with no concrete numbers ... 2

Multiple sclerosis and communication of side effects ... 4

Communication of resuscitation side effects with natural frequency and pictograph ... 6

Communication of cardiovasculair risk with risk table ... 7

Risk estimation together with patient for rare disease/infection with no concrete numbers

• Female<br>• Brother recently died through rare flesh-eating bacteria<br>• Doctor did not know concrete numbers<br>• Search for evidence in internet with patient<br>• Risk estimation together<br>• Shared decision
I have to think about case where a patient came to the practice with the story that her brother had died last week, that there was much uncertainty, because he had had a flesh-eating bacterium and had died of it. And via a message from certain family members it became evident that it is a dangerous bacterium and that prophylaxes had to be taken. And for her it was unclear whether she needed prophylaxes or not and what the risk was. [...] [In this case] I did not know the risk size. So the first step was clear: the patient had the idea that: “I quickly need antibiotics.” Because another family member got this advice through the GGD but they have not contacted me |the patient] yet. So she thought there was a big threat and that hurrying was needed and there was a need for prophylaxes. So I first need my own information... and I also informed the patient about that, because normally the GGD is doing a circle test. So then you explain the situation. And after I heard what contacts she had had and with which bacterium we are dealing with according to her, we called together the GGD, while she sat opposite to me in the consultation room. And we got clear that she closely failed to meet those criteria to get a prophylactic treatment. She also said: “Gosh, yes I understand that they make certain assessments.” Intensive contact from twenty-four hours onwards, that was the criterion, and she had had intensive contact in the last day but not twenty-four hours. And she said yes: “I may not be a housemate, but I have sat with my brother within a radius of a square meter, holding him, cuddling him, he coughed while I was there. And that while he was infected with the bacterium at that time, so I understand that they have cut-off points, for cohorts but yes I am an individual.” So I also understood her fear of: “Gosh I can got infected in those last 24 hours even though I do not meet the criteria of having stayed with the same person in a household for longer than 24 hours.” So then we looked together on the internet, when we got confirmed which bacterium it was and how it was transferable. Together we made an estimation, that you are not going to treat an entire cohort, but in her case, given her worry and her intensive contact, we felt that it would be wise to take those 3 tablets of antibiotics or 5 tablets of antibiotics. Afterwards she was called back, which was nice, also the GGD said formally you do not meet the criteria but considering your story we also think that it is better for you if you take it. So that was the risk assessment that was made. [...] you do not have a table for that, and it does not state how precise the contamination is, but what the consequences can be, so then you and

the patient will reason how important certain things are when do you know what to do, what do you do? are there other options, and then you make that assessment together, yes, what is the advantage, what is the disadvantage, that is three tablets of Zitromax gives little risk of side effects, and if the patient who is otherwise well healthy, and I think I have a lot of fear that I have sustained it then it gives so much rest to give those three tablets to the patient that it is preferable but then you are not concerned with risk tables, in the end it is of course also a risk assessment in your head , but not with hard numbers. [...] It's more that you and the patient decide together, you hear the fear, you hear the worry, you hear the wish and you combine that, you give that back to the patient. That is one side of the story. Well, this is the theory: “So theoretically, you do not meet the criteria. What are the options?” Watchful waiting, you might get symptoms fever, sore throat. [...] Or you would still despite that you do not meet the formal criteria, still can take those three tablets and you will weigh that against each other so I think that also falls under risk communication or under discussing alternatives. But I do not have a table for it that says: So and so many percent risk. And that is also because I do not know what the cut-off criteria are for the circle that is being treated with. [...] So then you mainly look at the individual case: What do I find understandable, what does the patient need, how do I estimate [...] what will be the best for that individual case. [...] And patient preference is one thing I think, but the other side is how do I estimate that the patient can handle this.

-- Interview C03 General Practice --

Multiple sclerosis and communication of side effects

- Multiple sclerosis
- Effectiveness of treatment options
- Communication of side effects
- Use of natural frequencies
- Use of bar charts

I would think about my time at the Multiple Sclerosis Day Clinic. Because there patients often have to make decisions that have long-term consequences, and we also have a bit more time than in the ambulance to talk about such things.
And often it's about the different immunotherapies that exist in multiple sclerosis. Just very briefly: multiple sclerosis is a nervous condition that affects in particular young people. And there are so-called immunotherapies, which can supposedly change the course of the disease. Especially so-called acute episode could be prevented. And most people think: I take a pill and then it works, so to speak, and then the acute episode does not occur. But if you look at the studies, then you can see that this is not true, so roughly let ́s say 10 to 40 % - depending on which immunotherapy it is - actually benefit from it and are actually acute episode-free, because they are taking this tablet. And I have printed an overview of all the medicines that exist. Fortunately, we have [this information] in our ambulance. And there are the absolute risks, the freedom of acute episode in people with placebo treatment and in people with the therapy of interest shown as bar charts. And then I take this sheet in my hand and I've copied it together myself so that then the side effects of the drugs are in the column next to it with so much and so much of a 100. And then I show it the patient and then say: “So, for you that and that drug come into question.” And then I cover the others and then I say: “So, now let's take a look. The drug here (then I tell first how they are applied. So there are injections, there are pills. This can already make a difference for the people.)” Then I explain how these bar charts are meant. Well, these are the patients who do not use drugs, but they think they are taking a medicine, but they do not actually take it. These are the patients who actually take a drug. And when you put it next to each other, you see that these patients here are the difference between the placebo and the therapy group, these are the people who really do not get an acute phase because they take the medicine. The others would have gotten anyways no acute episode. And then I explain that's 30 percent, then that's 30 out of 100. And then I turn that around and say: That means 70 would have anyways not gotten one. And then I'll go through all of this for every drug. And then I'll do the same with the side effects, but we just do not have those bar graphs for it. I then say: Do you already have a preference for a medication? And then I start with this one. And then I say: Well, in so many people this side effect occurs, in so many people that side effect occurs. And then I try to say something like: A needle puncture reaction is not so bad. That may occur in more people, but that - I have to say that may not be really scientific - but then I'll just say: That's not such a bad side effect. Or I say something like: Oh for women hair loss is always very bad, but that's not so bad in reality. If you look at pictures on the Internet, it shows that the hair fells out in a certain growth phase and then it grows again. That takes only a few months. So I relativize it for some side effects a bit. And then there are some side effects, which are very rare, but quite dangerous. And

I always say those at last. Because, most of the time one patient finds nausea worse than hair loss and the other one just the other way around, but everybody thinks it's bad to die. So I say: And now there's a side effect that – do not be afraid that sounds very scary now, it is very rare, but it's very dangerous and you have to know that it exists. And in XX of 1000 people can an encephalitis occur, which can even lead to death. But there are ways and means to ensure that people are not affected by it. In this case there is a blood count that you can control.

-- Interview C11 Neurology --

Communication of resuscitation side effects with natural frequency and pictograph

Case resuscitation

- advanced care planning and whether or not to resuscitate the patient
- use of population pictogram
- use of natural frequency

So, with all the patients I try to make a non-resuscitation agreement and then I discuss with patients - I start with: "If you could tell me what your ideal death would be, what would it be like?" And most patients tell me: "Well, I want to grow old and when I'm very old I want to go to bed in the evening and not wake up in the morning."
And I want them to tell me that, because it's kind of a cue for me to start talking about resuscitation and then I say: "Well, do you know what happens when you go to bed and don't wake up in the morning?" And most patients say: "Yeah, well, I die." "Yeah", I say - "okay, but do you know what happens? Why you are dying?". Most patients say: "Well, I don't know." And then I explain them that when they go to bed and don't wake up in the morning, most likely they suffered from a heart attack or a cardiac arrest, and that is a tremendously preferred way of dying. And: "What would you like me to do if you're not in bed?! It's not that you're not waking up, but what when your heart stops beating, when you get a heart attack or a cardiac arrest. What do you want me to do? Do you want me to resuscitate you?". And then the patient says: "Why are you asking me that?" I say: "Well, I ask you that because I would like to discuss with you what we need to do when something like that happens, because I don't want that I am going to resuscitate you and that you later tell me: "Well, I didn't want that, because it wasn´t my therapy for me it was my time to go." So and then they ask me: "But, what are the chances of survival with resuscitation?" And then I take my chart, it's a square, within it I think about 40 or 50 pictograms of people and if we resuscitate 100 persons over 75 years approximately 8 will survive and 92 will die, and of those 8, 4 will survive with little or no late effects and 4 will survive but will have severe neurological or other organ damage. That's what I use as an example, and many people tell me that. "Well, are those chances really that low when you are being resuscitated?" And then I explain to them: "When you are young, and the cardiac arrest or heart attack is the only problem you have and somebody watches you having a heart attack, then the chances are relatively good. But when you are old, like you are, and you have not only a problem with your heart or your blood vessels but you have also other chronic diseases...", (which apparently are present in these patients which I'm discussing this with) then the chances are decreasing with age, with other conditions. And that's why the chances are so low." And then I explain to them: "I don't expect you to have a heart attack now or in the immediate future, but I just want to know what to do if the possibility that if it happens what we should do. And many people, after this risk communication, decide that they do not want to be resuscitated. And some say: "Well, I know it's a really small chance of a good life after a resuscitation, but I am really attached to life so I want to have everything done that is possible and I do want to be resuscitated." And that's the minority.

-- Interview C02 General Practice -

Communication of cardiovasculair risk with risk table

- Patient with a 100% gallstone
- Illustration of cardiovasculair risk with risk table
- Low literacy

I can remember a patient who came to my practice and who said: ”I was operated on a gallstone and that gallstone was 100% cholesterol.” So at that moment the patient asked me: "Well, does that matter to me, or am I ready now?" No, I say, it would be wise to visualize your cardiovascular risk, so I started to measure his weight, his abdominal circumference, his blood pressure and his LDL cholesterol.

And what risk communication entails among other things, I think, is at least talk about what it means. Because you want that A: Not to make people unjustly into patients, because if you feel as a patient then you are no longer as happy as you were before. And B: You want to be able to say because you have a slightly higher blood pressure and you have had a cholesterol stone, you have - normally a risk of a heart attack is one percent in your age, but because you have those other [risk] factors, the risk rises to three percent or four percent. Anyway, noticed that if you say one percent, two percent, three percent, that is still not very clear. So in that example I do not use percentages, but then I use, in this case I used a card that the Dutch family doctor association made, that is red, orange, yellow, green, a traffic light (NHG standard).

So then I say like: “Well you have red, orange, yellow, green and most people are here (pointing on yellow). But because you have had that history with that gallstone that is one hundred percent cholesterol, which is normally not the case, you put yourself in that middle area. Then they ask: "Well what does that mean doctor?" Well that means that if you were in red you'd have to take all kinds of medication, but because you have a slightly increased risk, so you are in the orange traffic area, that means I have to tell you to some lifestyle changes then I tell things like: no smoking, moving, exercising. What I then also sometimes do is, I show them the risk table. But I notice in my practice, especially with the lower educated, that they do not understand it well. So I usually do this: These are 100 people from my practice. And, if you're in the green field, those people have the chance to have a heart attack someday. Now that you are in that orange field, you have an extra square. So I try to make it as visual as possible. Without too many numbers and things. So that's how I try to do that. So that's really just explaining where someone stands. And my experience is that, certainly when I look at the past, it has been emphasized too little. We are actually moving pretty quickly from risk communication to these are your options and yes what will we do. So continuing too fast in that circle of shared decision making. While if we do this [risk communication], if people understand this well, I think they are more receptive to those next steps.

-- Interview C04 General Practice --
