# Supplementary material for: A qualitative exploration of clinicians’ strategies to communicate risks to patients in the complex reality of clinical practice
Source: PLoS One. 2020 Aug 13;15(8):e0236751. doi: 10.1371/journal.pone.0236751 (PMC7425874; doi:10.1371/journal.pone.0236751)
Supplement: S3 Appendix — (PDF) [file pone.0236751.s003.pdf]

Table of Contents

S3: Risk communication strategies and narratives *2*

Framing **2**

Verbal Risk Communication **3**

Numerical Risk communication **4**

Visual Risk Communication **6**

Self-drawn drawings 6

Existing tools 8

Uncertainty **10**

S3 Appendix: Risk communication strategies and narratives

Framing

Framing strategy	Narrative of Strategy
**Positive and negative Framing**	In general I am direct and I prefer to say the chance is 1 in 10 that you will die. [...] But I vary this [framing] a bit. And that also depends on, well I think it really depends on the situation in which that patient is at that moment, and also a bit on where I think it is good to put more emphasis on. [...] For example recently in the department someone said: 'oh I ́m afraid that next month I will have the disease back' although I knew that the chance was very small that the lady would have the disease back in the following month. But it wasn't zero. And if I told that lady 'you have the chance of one in ten that you will have the disease back next month', she would have been worried the whole evening, while if I told her 'well the chance is nine in ten that it just goes well 'then I have reassured that lady. Then I actually use the risk communication [...] framed to the patient to also support that patient a little bit.<br>-- Interview C08 Internal Medicine – Hematology –<br><br>Yeah I think that also depends on the case. For example in oncology patients I see that oncologists in the hospital, many oncologists always talk about survival. You have a 22 percent or 12 percent chance with this fourth line chemotherapy that you will live a little bit longer. Then patients come to me in my general practice and they want to discuss it with me and then I ask what they think is important quality of life or quantity of life. And they tell me well my quality of life is more important and I think I'm going to talk about the chances of death. If they say: Well I want to live as long as possible. Then I think it's more framed like in, in, in terms of survival. So it really depends on the case. And It really depends on the goals of the patient. I think that's really the most important thing. Their goal is survival, I guess I will use those terms. If their goal, if they talk about avoiding death I think I am using the term death rate. It really depends on the patient.<br>-- Interview C02 General Practice --<br><br>So, that varies a little bit per patient, and if you notice that a patient becomes very anxious through your risk communication, then you also nuance that. Then you try to make the risks a little bit smaller because you have the idea that a patient becomes too frightened because of the risks. So, you also adjust your style of risk communication somewhat.<br>-- Interview C15 Orthopaedics --<br><br>I am inclined to reasoning it towards the healthy side. In case of cardiovascular risk, so if people are smoking or something else, I say people who have the right behaviour and who do not smoke and have normal blood pressure, from those four in 100 get a heart attack and if you continue like that then you belong to the group in which eight in hundred get this. So, then I am more inclined to argue towards the disease.<br>-- Interview C04 General Practice --

Verbal Risk Communication

Verbal RC Strategies	Narrative of Strategy
Use of metaphor<br>• Weighing scale, chance to get son or daughter	And then it seems for the people in the end often as if it is almost certain that they have that deviation. So at the end of the conversation I say to those people, “It now seems like that percentage or the chance/odds that you have that deviation is very high, much higher than the chance that you don't have it, because I had to tell you a lot about it. While if you do not have the deviation, you do not have an increased risk, you may not have passed it on to your children for example.” And then I always show it is like a weighing scale: So then I go up with one hand, and down with the other hand, I go to the same height and then I say, "It's a 50 % chance that you are a carrier, 50 % that you are not a carrier. So it's 50:50." I also often say, “Just as likely to get a son or a daughter in a pregnancy.”<br>-- Interview C14 Clinical Genetics –
• Umbrella	If you look at the cloudy sky, and you ask ten people if they want to take an umbrella or not, then one part will always take an umbrella, one part never, and one part depending on how cloudy the sky is. [...] But it is not about whether I would like to bring an umbrella, because it is about, “Do you want to bring an umbrella?” And that sometimes we would decide differently with the same data [...] Everyone perceives risk differently, and one person might not mind walking around and carrying such an umbrella the whole day for nothing, because she definitely doesn't want to have damaged her new haircut. And the other person thinks 'I'm really not going to walk around all day with such an umbrella.' This person finds the disadvantages of that umbrella much greater. And with that metaphor I think you get people to understand this better.<br>-- Interview C05 Gynaecology –
• Picture of a belt	So, sometimes I have the impression that the patients don ́t understand what ́s going on what ́s going to happen with them. And I compare it sometimes with a with a belt. They are on the belt and then you do all kinds of diagnoses and at the end of the belt is the operation table.<br>-- Interview C09 Head and Neck Surgery – Oncology --
Use of comparision<br>• Lottery	Sometimes also to illustrate how big the chance is to get something, because the numbers are very abstract. “How big is the chance that you will win the lottery?” If you compare that with the chance of a side effect, or the chance of getting a disease if you have a certain predisposition. “How many people do you know who have won a million in the lottery?”, something like that.<br>-- Interview C03 General Practice –

Numerical Risk communication

Verbal RC Strategies	Narrative of Strategy
Use of natural frequency	Then I say: And now there is a side effect that, do not be afraid, it sounds now a bit scary, but it is very rare, but it is very dangerous and you have to know that it exists. Namely in in XX of 1000 people, a [...] encephalitis can occur, which can even lead to death. But there are ways and means to ensure that people are not affected by it, and in case it occurs there is a blood value that you can control. -- Interview C11 Neurology --<br><br>I once learned [...] that 5 in 100 is easier to understand than 5 %. So, I always try to do that. - Interview C08 Internal Medicine - Haematology --<br><br>1 out of every 100 patients we operate get an infection. -- Interview C15 Orthopaedics --<br><br>And then I explain that's 30 out of 100. And then I turn that around and say: That means 70 would have anyways not gotten one. And then I'll go through all of this for every drug. -- Interview C11 Neurology --<br><br>[...] And those who really got the medication, so in the treatment- group were 100 patients. From those 28 got a boost and 72 got none. -- Interview C12 Neurology –
Use of absolute risk	Well, so I'm basically always explain them - if I - I ask the patient: Have you ever heard of absolute numbers or absolute risk reduction and relative risk reduction? And then quite a lot of people who are involved with statistics or something like that they say: Yes, it tells me something. And those who do not have that then say: No, I haven´t heard of it. And then I say to the patient (then we have these bar graphs) where now let´s say in relation to the one drug: that there are 100 patients represented and now of the 100 patients – (and that is now the bar chart in relation to freedom from acute-phase) - so all the drugs that are admitted are two years, sort to speak, tested in studies and tested in terms of freedom from acute-phase. And if I have a drug now, say Tecfidera, the results of the study are so that I can say: so, I have 100 patients and 100 patients have taken placebo for two years and 44 of them still got a boost and 56 did not get one. That's kind of how I start. And then those who really got the drug, so in the verum group were the 100 patients. 28 got a boost and 72 got none. 72 patients from 100 did not get a boost (acute phase) under the medication Tecfidera. That is quite good, but we have to subtract the 56 patients from the placebo-group since they did not take medication. So statistically speaking we are left with 16 left in the middle sort to say who have an effect based on the medication Tecfidera. In relation to the boost within two years.<br>-- Interview C12 Neurology --
Use of NNT	For example in patients with a upset stomach, then I say, “Well ok now you have those stomach problems and that goes on and on, and now we have shown that bacteria in your stomach. And we know with stomach ulcers, but

	you don't have that, if we treat that bacterium then the stomach ulcer will pass, but you have stomach complaints without a stomach ulcer but the bacterium. Then we know from all kinds of studies that you have to treat 27 people with treatment to kill that stomach bacteria, in order to cure 1. So we can do it, but then you know, if you have 27 people, one of them [will be cured], and maybe you are the lucky one." So kind of description of number needed to treat I do then.<br>-- Interview C04 General Practice –<br><br>"Well, if I treat 100 patients, in 100 patients there will be no blood clots." [...] We need to treat 100 patients and in 5 or 6 patients in the next 10 years, we will be able to prevent [the event].<br>-- Interview C08 Internal Medicine Haematology --

Visual Risk Communication

Self-drawn drawings

<table>
<tr>
<th>Diagram of mortality in leukemia patients in relation to stem-cell transplantation with explaining narrative</th>
<th>Diagram peak age of breast cancer with explaining narrative</th>
</tr>
<tr>
<td>

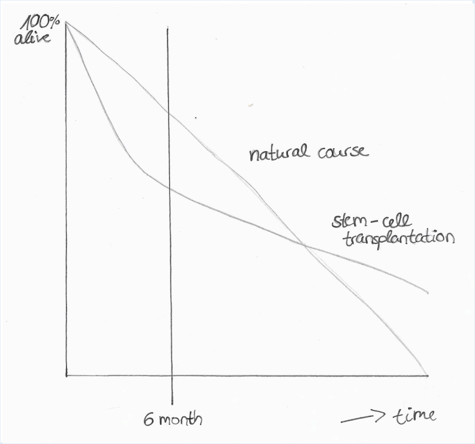

100% of people are alive on the vertical axis. And [on the y axis] 0% are alive. Then of course everyone starts at the top at 100%. And the natural course of the disease is that people usually die one by one and then in time they all eventually die from that disease. With the stem cell transplantation more people die early on, so you get a steep curve down, but it then bends, and somewhere it crosses the other curve. And that is something that I find difficult to explain verbally; it is easier to say that you have more risk of dying here in the first six months or so. But ultimately, in the longer term - say two years or so and often two or three years - that you have a greater chance of survival.
-- Interview C08 Internal Medicine Hematolog--

</td>
<td>

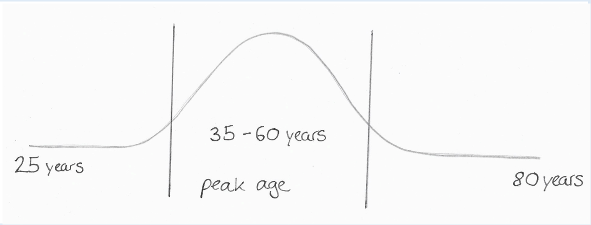

So what I do is: make a line like that and then I say here comes a peak age and then it goes down again. Here at the beginning of that line a woman is 25 years old, at the end she is 80 years old. And that peak age - it is between 35 and 60 years. Now I know that people sometimes don't fully understand that well, so then I say afterwards: "that means that most women who are carriers of this abnormality will have breast cancer during this period, at that peak."
-- Interview C14 Clinical Genetics --

</td>
</tr>
</table>

Illustration of treatment effect with NNT

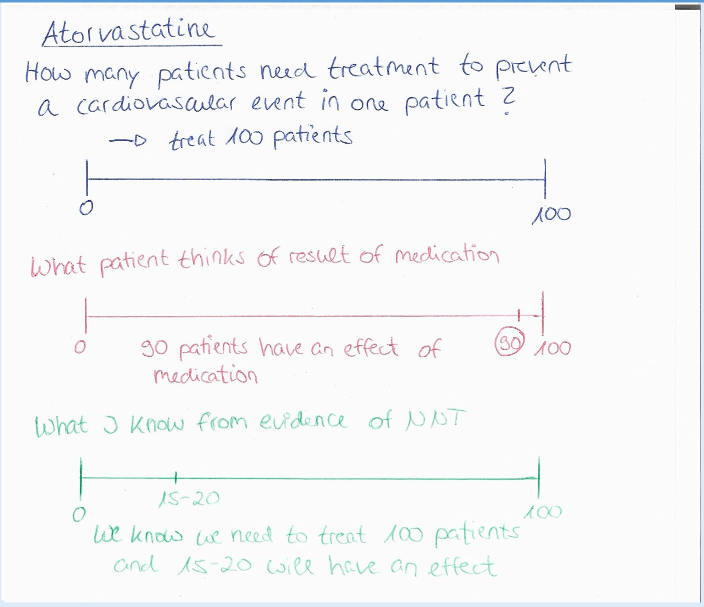

I draw a line from zero to a hundred and with how many cases of blood clots in a vessel do you think we will diminish with starting these anti-cholesterol tablets? And then patients always think "Well, if I treat 100 patients, in 100 patients there will be no blood clots." and then I draw [...] "Well, actually it's really difficult, we need to treat 100 patients and in 5 or 6 patients in the next 10 years, we will be able to prevent [a blood clot].
-- Interview C02 General Practice --

Existing tools

Visual RC Strategies	Narrative of Strategy
**Use of existing tools**<br>• use of pictograph	I say, "Well, I ask you that because I would like to discuss with you what we need to do when something like that happens, because I don't want that I am going to resuscitate you and that you later tell me, "Well, I didn't want that, because it was my time to go." So and then they ask me, "But, what are the chances of survival with resuscitation?" And then I take my chart, I have this, it's a square, which within it I think about 40 or 50 pictograms of people and if we resuscitate 100 persons over 75 (years), about 8 will survive and 92 will die, and of those 8, 4 will survive with little or no late effects and 4 will survive but will have severe neurological or other organ damage.<br>-- Interview C02 General Practice –
• use of CVRM scheme/NHG	Yes, the NGH standard is the risk table. So then I say like: "Well you have red, orange, yellow, green and most people are here (pointing on yellow). But because you have had that history with that gallstone that is one hundred percent cholesterol, which is normally not the case, you put yourself in that middle area. Then they ask: "Well what does that mean doctor?" Well that means that if you were in red you'd have to take all kinds of medication, but because you have a slightly increased risk, so you are in the orange traffic area, that means I have to tell you to some lifestyle changes then I tell things like: no smoking, moving, exercising. What I then also sometimes do is, I show them the risk table.<br>-- Interview C04 General Practice –<br><br>And then I look together with the patient at the table and then I show: This is the average of if you have 100 people in a row, of those 100 people you see that if those people have this blood pressure and these cholesterol values [...], you see that the risk profile decreases "so much" when you stop smoking. But if you additionally ensure that your blood pressure gets lower, you see that of those 100 people that "so much" eventually get the disease or not. So, then you use that table to show the patient how he can

	improve his own risk profile. -- Interview C03 General Practice –
• use of bar charts	And then I explain how the bar charts are meant. Well, these are the patients who do not use drugs, they think they are taking a medicine, but they do not actually take it. These are the patients who actually take a drug. And when you put it next to each other, you see that these patients here - so the difference between the placebo and the therapy group - these are the people who really do not get an acute episode because they take the medicine. The others would have gotten no boost anyways. -- Interview C11 Neurology --<br><br>Well, so I'm basically always explain them - I ask the patient: Have you ever heard of absolute numbers or absolute risk reduction and relative risk reduction? And then quite a lot of people who are involved with statistics or something like that they say: Yes, it tells me something. And those who do not have that then say: No, I haven´t heard of it. And then I say to the patient (then we have these bar graphs) where now let´s say in relation to the one drug: that there are 100 patients represented and now of the 100 patients – (and that is now the bar chart in relation to freedom from acute-phase) - so all the drugs that are admitted are two years, sort to speak, tested in studies and tested in terms of freedom from acute-phase. And if I have a drug now, say Tecfidera, the results of the study are so that I can say: so I have 100 patients and 100 patients have taken placebo for two years and 44 of them still got an acute episode and 56 did not get one. That's kind of how I start. And then those who really got the drug, so in the treatment group were the 100 patients. 28 got an acute episode and 72 got none. 72 patients from 100 did not get an acute episode under the medication Tecfidera. That is quite good but we have to subtract the 56 patients from the placebo-group since they did not take medication. So statistically speaking we are left with 16 left in the middle sort to say who have an effect based on the medication Tecfidera. In relation to the acute episode within two years. -- Interview C12 Neurology –

Uncertainty

Uncertainty	Narrative of strategy
Verbal communication of aleatoric uncertainty	But a few get it a bit earlier,and a few get it a bit later or not at all because the risk is also not 100 %.<br>- Interview C14 Clinical Genetics –<br><br>10 patients who swallow [a medication], then 1 of those 10 gets problems from it, and we cannot predict in advance who that is.<br>-- Interview C03 GP --
Giving a range	Yes, when it is about time I find it very unpleasant to give a defined time. Because it just isn't there. We only know an average of 100 comparable patients. [...] We never know for the one person how long it will take. [...] To give them a bit of an idea roughly, and the reason I don't mention the exact number is that I know that there is variety in it with different patients, and if you give a number then people often keep on holding on to that one number. [...] In this case, that is my assessment as a clinician, I find it not useful that the people know exactly a number what exactly the average survival is. [...] the idea of where it goes towards [is more important for them]. And when people ask me: `What is the average survival, can you say that?´ Well then I have to mention six months but in the beginning I try not to do that.<br>- Interview C08 Internal Medicine Hematology -<br><br>[...] you never know. I have seen patients with chance of 95 % for cure. And they die. And the other way around, patients incurable and they survive. [...] You never know. [...] But if you are honest. You say: [...] if you have 1000 patients [...]. 90% will survive or 90% will die. That´s honest. But then again that doesn´t say anything about you. We don´t know. That´s also the problem with how long do I have? [...] doctors who say: Ok you have [...] three month because if you have 1000 patients with the same disease, one patient will survive one hour and one will survive 10 years and the mean is three month. [...] But I never use these kinds of indications. Because it´s I think it´s harmful. Because I had patients and then they came to me in in May and they: Ok, I was with the medical Oncology and they said I have two month to go and so... And then [they think] it´s the last visit. And then they said goodbye. And this lady [...] she came back in December. And still in a good condition. And we were just a bit talking and [she] said yeah: The problem is I gave all my stuff for Christmas away. What shall I do? I say: Yeah, you have to ask it back. [...] And she died next year in in June.<br>-- Interview C09 head and neck surgery –
Verbal communication of epistemic uncertainty	Yeah, I communicated it [uncertainty] a little bit more open. I say for a lot of disease or in your case we don't know exactly what is going to work because there isn't a lot of research supporting therapies or treatments for your disease or for your case. So we need to have trial

and error what is working for you. -- Interview C02 General Practice --
