# Supplementary material for: A qualitative exploration of clinicians’ strategies to communicate risks to patients in the complex reality of clinical practice
Source: PLoS One. 2020 Aug 13;15(8):e0236751. doi: 10.1371/journal.pone.0236751 (PMC7425874; doi:10.1371/journal.pone.0236751)
Supplement: S4 Appendix — (PDF) [file pone.0236751.s004.pdf]

# S4 AUDIT Trail Qualitative Content Analysis

In the following the planning/preparation, organizing (deductive and inductive analysis) and resulting phases in the content analysis are going to be presented. The qualitative content analysis is based on the framework of Elo&Kyngäs 2008.

## Planning process/ Preparation Phase (Elo&Kyngäs 2008)

| | |
|---|---|
| Paradigm, Ontology, Epistemology and study design | 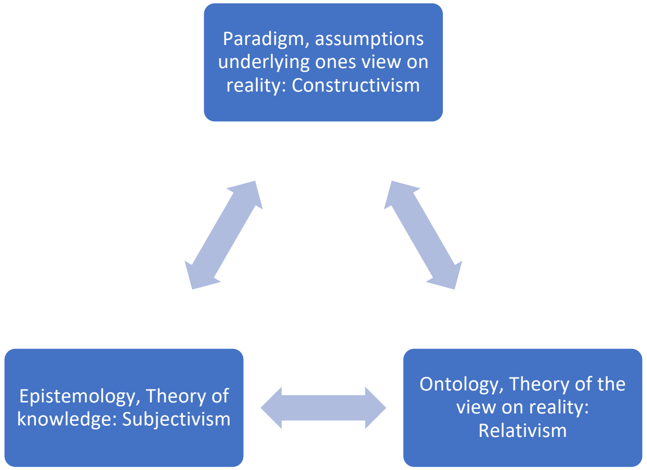 <br>Choice of research paradigm with the ontological and epistemology orientation was carefully considered after reading of important literature and discussion within the research team. Likewise, the study design was carefully chosen considering the aim of the research (Bergmann 2012, Bradshaw 2017). |
| Aim of the study and sample | The qualitative descriptive study aims to gain a deeper understanding of clinician's actual strategies in communicating risk in daily clinical practice. A comprehensive insight into the best practice approaches of a sample of clinicians who have experience of, or are at least familiar with, the concept of shared decision making (SDM), may provide valuable examples of real life risk communication strategies, which could support young doctors in the process of acquiring SDM skills (Giroldi 2017). To promote the development of risk communication |

| | |
|---|---|
| | training for young doctors, we explored the strategies used by clinicians to communicate risk to patients and aimed to extract illustrative examples (narratives) and visualizations of these strategies.<br>In qualitative studies sample sizes are rather small, usually between 1-30 participants (Bengtsson 2016). In order to answer the research question with sufficient confidence we sampled clinicians that are known to be exposed to communicate risk on a regular basis in daily clinical practice. In order to obtain a broad spectrum of perspectives, sampling was based on gender, age, experience and clinical field. Sampling was part of the iterative process of data collection and analysis and was stopped once data saturation was reached. |
| Data collection method<br>- How do I collect the most suitable data for my content analysis? Is this method the best available to answer the target research question?<br>- Should I use either descriptive or semi-structured questions?<br>- Self-awareness: what are my skills as a researcher?<br>- How do I pre-test my data collection method? (Elo et al. 2014) | We conducted semi-structured interviews as this approach is considered the most suitable to elucidate clinicians risk communication strategies and the researcher has the opportunity to deepen the discussion (Bengtsson 2016). The interview was semi-structured since the questions are partly informed by a conceptual framework based on a literature review on risk communication strategies/recommendations/best practice approach.<br>I am a young, ambitious researcher at the beginning of my career. During the conducting phase of the qualitative study, I was still in my master program. However, the master's program "health sciences research master" prepared me well in various methodological, and analytical issues and skills for conducting research. The course in "qualitative research" and the thorough preparation phase of the qualitative study by means of reading crucial literature and writing a research proposal allowed a steep learning curve in improving my qualitative research skills. Further, I conducted two pilot interviews to practice my interviewer skills. I informed myself thoroughly about qualitative content analysis (Bengtsson 2016, Bradshaw 2017, Elo&Kyngäs 2008). Every step of the research project was under guidance of an experienced senior researcher (TW) and additionally an experienced qualitative researcher (EG) supported the data analysis process. |
| Choice of analysis method | Qualitative content analysis is a content-sensitive and flexible approach to analyze qualitative data (Elo&Kyngäs 2008). It can be used in a deductive and inductive way. The qualitative content analysis process was based on the method of Elo&Kyngäs 2008, which also outlines the combination of deductive and inductive content analysis (Elo&Kyngäs 2008). |

| | |
|---|---|
| Selecting the unit of analysis | The full interview transcript was analyzed and words to sentence were chosen as the unit of analysis. |
| Making sense of the data | In order to became immersed in the data, transcripts were read and the following questions were clarified: Who is telling? Where is this happening? When did it happen? What is happening? Why? (Elo&Kyngäs 2008). |

## Organising Phase (Looking's 2008)

### Deductive Content Analysis

<table>
<tr>
<td>Developing Analysis Matrix</td>
<td>
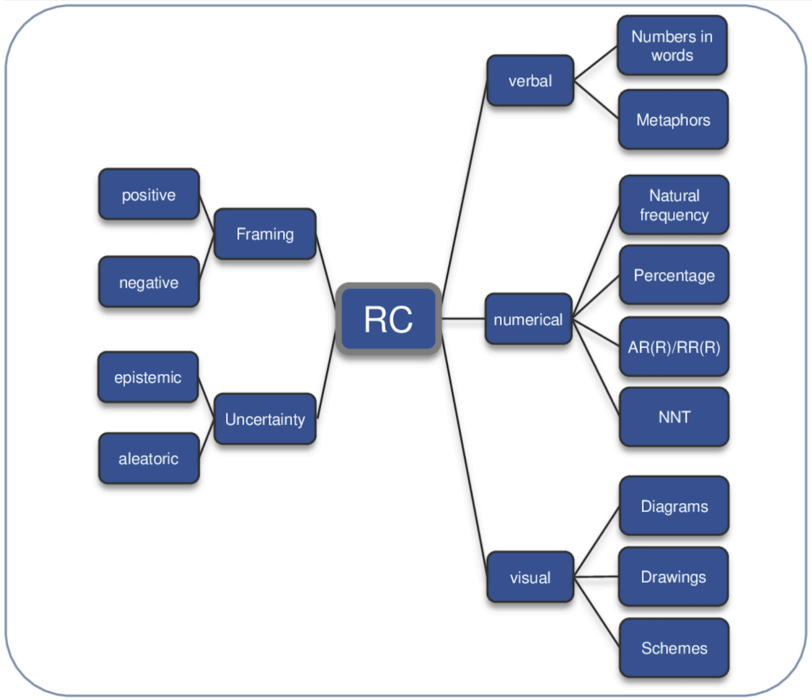


**Fig 1 Framework risk communication (RC)**

**AR(R)= Absolute Risk (Reduction), RR(R)= Relative Risk (Reduction), NNT= Number Needed to Treat**
</td>
</tr>
</table>

| | |
|---|---|
| | Based on the deductive conceptual framework established in the literature review (Fig 1), we started with a deductive content analysis to gather risk communication strategies according to the categories described in the framework. |
| Data gathering by content | Gathering of risk communication strategies according to the framework. Use of abbreviations: S.= Strategy; SS.= Sub-strategy; NS. = No strategy<br><br>*Example:*<br>**S. use of numerical RC**<br>• SS. use of AR preferred<br>• SS. use of natural frequency<br>• SS. use of percentage<br>• NS. only use of words<br>**S. use of visual RC**<br>• NS. no use of drawings<br>• SS. using diagram from patient file with kidney function<br>• SS. drawing diagram lifetime risk + explanation<br>• SS. using pictogram<br>• SS. drawing of recessive disorder<br>• SS. using of lego bricks<br>• SS. use of diagram population risk<br>**S. use of framing**<br>• SS. use of neg framing<br>• SS. use of pos framing<br><br>After application of the conceptual framework (Fig 1) in the coding procedure it became evident that the framework was not sufficient enough to capture all findings. For this reason, next to the deductive approach guided by the conceptual framework (Fig. 1) an inductive approach was collaterally initiated to capture further risk communication strategies as well as important content and contextual factors on clinician and patient and consultation level. |

## Inductive Analysis

| | |
|---|---|
| Open Coding process | The transcripts were read open-mindedly and important text passages (meaning units) were highlighted. Headings and notes for those identified meaning units were written down in the transcript. Transcripts were always independently coded by two researchers (RR and EG or RR and TW) and discussed.<br><br>Examples:<br>dilemma awareness versus anxiety<br>frustrated doctor<br>patient is not emotionally prepared<br>health literacy<br>patients do remember the explanation but do not relate message to themselves<br>confronting the patient with consequences<br>what is the goal of risk communication<br>check for patient understanding<br>aleatoric uncertainty difficult to explain in retrospect<br>type of patient<br>severity of risk<br>risk communication depends on patient preference<br>dilemma quality vs quantity of life<br>goals and wishes of patients are core of shared decision making → decrease in RC |
| Coding Sheet | After open coding the codes were gathered in a coding sheet. Categories were freely generated at this stage based on the open coding procedure. The following abbreviations and explanation of codes were used to broadly categorize codes:<br><br>S. → main strategy<br>SS. → sub-strategy<br>NS. → no strategy<br>CF. → context factor<br>D. → dilemma<br>W. → wish<br>C. → Content/types of risk, cases<br>Narr. → example narrative |

| | |
|---|---|
| | *Examples for summarizing codes identified during the open coding process in a coding sheet with broad categorization:*<br><br>**Content/types of risk=C**<br>• Types of risk (prevention, treatment option, treatment (side) effects, screening test, genetic disposition)<br>• prognosis when do I have so start dialysis<br>• Cases<br>  ○ C. women with breast cancer heredity of 2 disorders<br>  ○ C. case carrier<br>  ○ C. case unclear case for prophylaxes (flesh eating bacteria)<br>  ○ C. older women and breast cancer<br>  ○ C. PSA screening pos test<br><br>**Doctor´s strategies of RC= S. and SS.**<br>main strategies + sub strategies<br><br>• SS. Risk estimation together with patient for rare infection<br>• SS. trade-of off between pros and cons<br>• SS. test validity - put test in perspective<br>• SS. Acknowledge lack of knowledge - search together<br>• SS. transparency over deliberation thought of doctor<br>• SS. trade-off deliberation<br>• SS. acknowledge lack of knowledge<br>• N.SS. steering of people<br>• SS. adjust to patients pers. situation<br>• SS. being honest<br><br>• SS. longer treatment duration detailed comm of side effects<br>• SS. short-term and small risk no detailed side effects<br>• SS. num risk + consequences treatment burden<br>• SS. relating small % to experience with other cases<br>• S. comm of most important facts<br>• S. 1. global talk 2. tailored to test result<br>• S. give sources for further info<br>• S. split talk if too much info<br>• S. find right moment for RC |

| | |
|---|---|
| | • S. telephone consultation<br><br>**Dilemma/ challenges= D**<br>• D. awareness of risk vs. not worrying patient<br>• D. treatment burden vs size of risk<br>• D. QoL vs side effects<br>• D. necessity treatment vs. treatment burden<br>• D. QoL vs. life expectancy<br>• D. trade-off quality vs quantity<br>• D. information overload vs. anxiety<br>• D. neg test vs hereditary regardless<br>• D. preventive treatment vs. remaining risk<br>• D. describing risk vs. worrying patient<br>• D. transparency over side effects vs. fan fear<br><br>**Context factors/ influencing factors= CF.**<br>• **Patient factors**<br>  ○ CF. patient type<br>  ○ CF. health literacy<br>  ○ CF. patient emotions<br>  ○ CF. patients do forget information partly<br>  ○ CF. many patients do not want further info - ostrich strategy<br>  ○ CF. different in men and women<br>  ○ CF. patient condition and information load<br>  ○ CF. age of patient<br>  ○ CF. ability to understand  risk in this moment<br>• **Doctor factors**<br>  ○ CF. motivation<br>  ○ CF. expertise of doctor<br>• **Size of risk**<br>  ○ CF. high risk<br>  ○ CF. small risk |

*Insight into QUIRKOS Software Program: Single codes.*

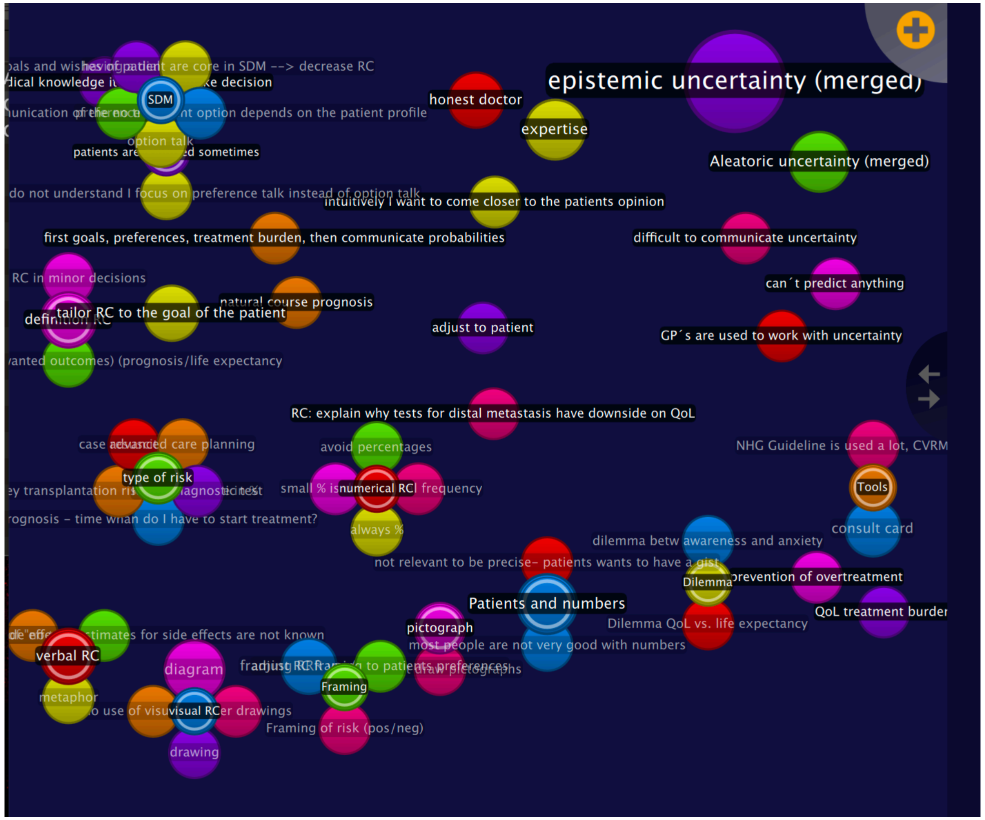


Many codes are generated through inductive coding process as can be seen in the screenshot of the QUIRKOS program. The bubbles represent the single codes and can subsequently be grouped. Coding of interview transcripts happened iteratively during data collection and analysis process. Checking the codes repeatedly was needed. Some codes changed as the study progressed.

<table>
<tr>
<td>Grouping process</td>
<td>In the grouping process the codes were grouped under higher order headings to reduce the number of single codes as illustrated in the following picture (Elo&Kyngäs 2008):<br><br>
Example 1 grouping of codes referring to dilemmas/challenge’s clinicians face<br><br>
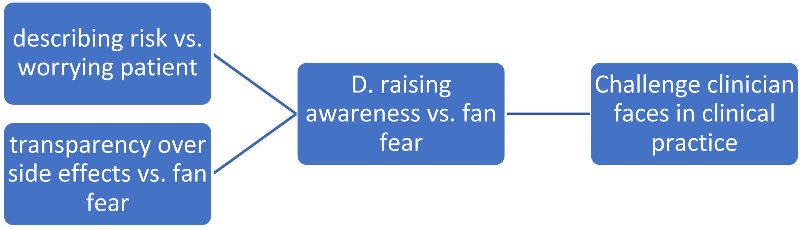
<br><br>
Example 2 grouping of codes that show a goal of clinician<br><br>
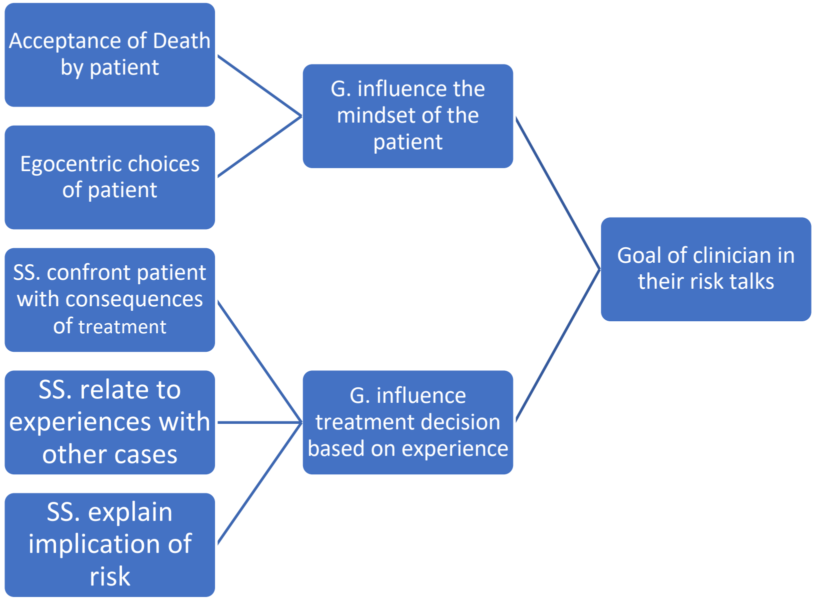
</td>
</tr>
</table>

*Insight into QUIRKOS Software Program: Grouped codes*

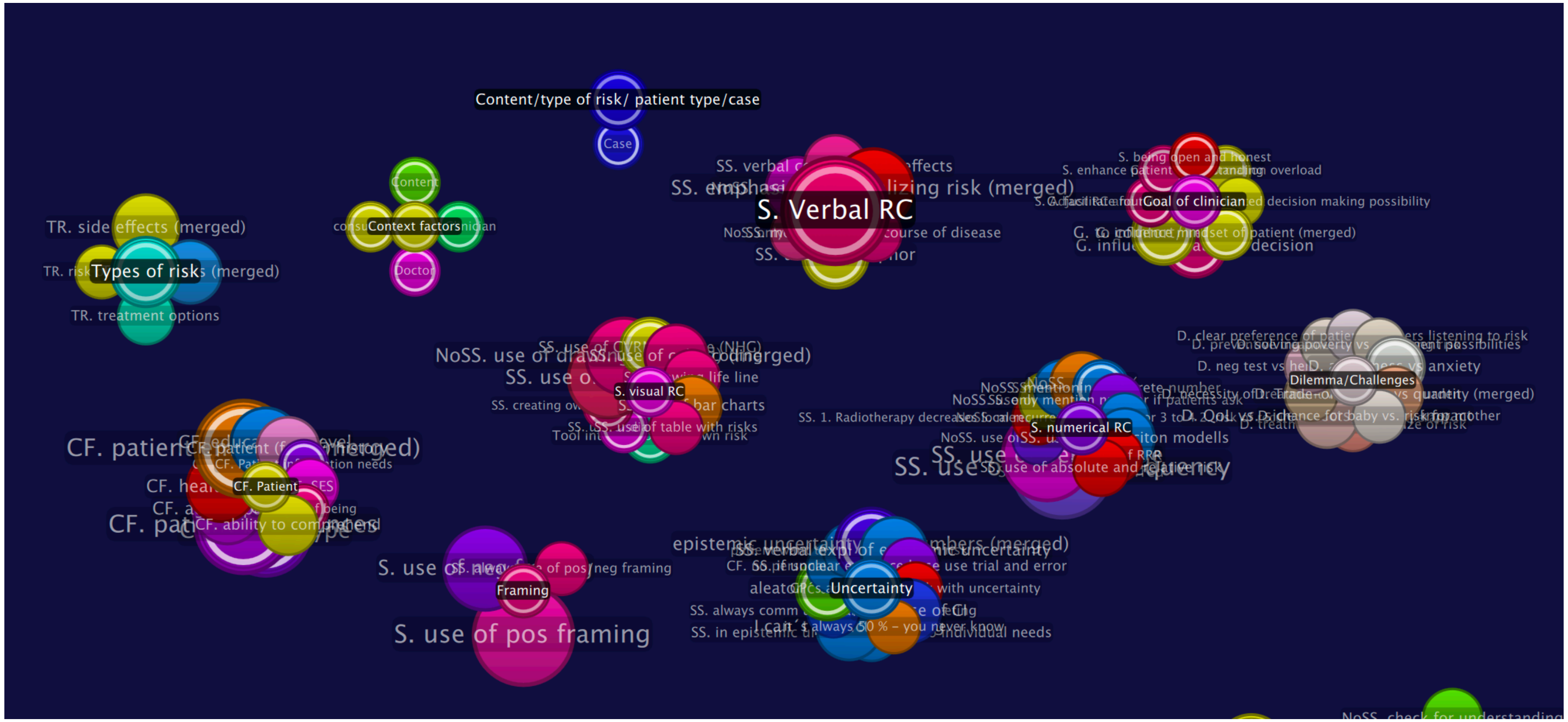

| | |
|---|---|
| | *Example QUIRKOS program: Grouping of codes referring to dilemmas/challenge's clinicians face in clinical practice* 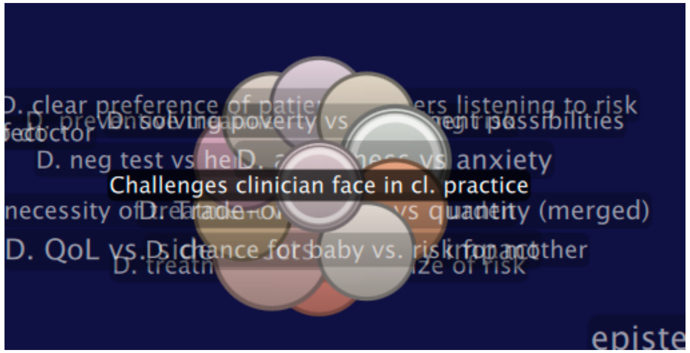  |

| | |
|---|---|
| | 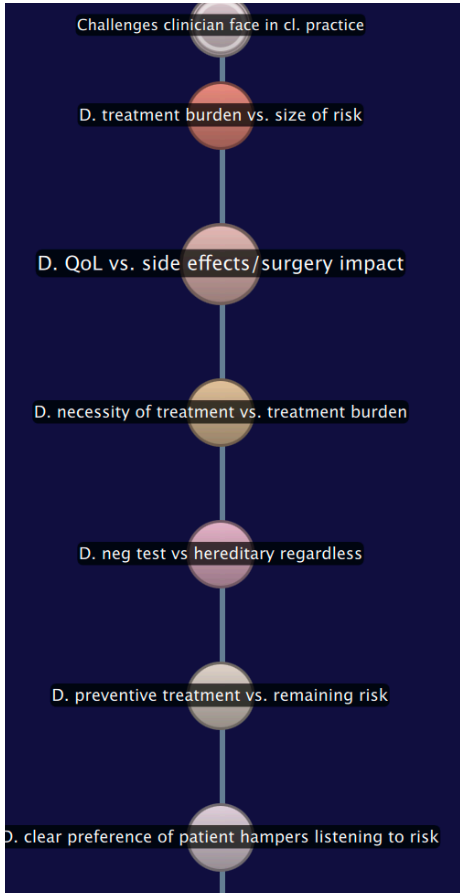Challenges clinician face in cl. practice<br>D. treatment burden vs. size of risk<br>D. QoL vs. side effects/surgery impact<br>D. necessity of treatment vs. treatment burden<br>D. neg test vs hereditary regardless<br>D. preventive treatment vs. remaining risk<br>. clear preference of patient hampers listening to risk |

| | |
|---|---|
| | 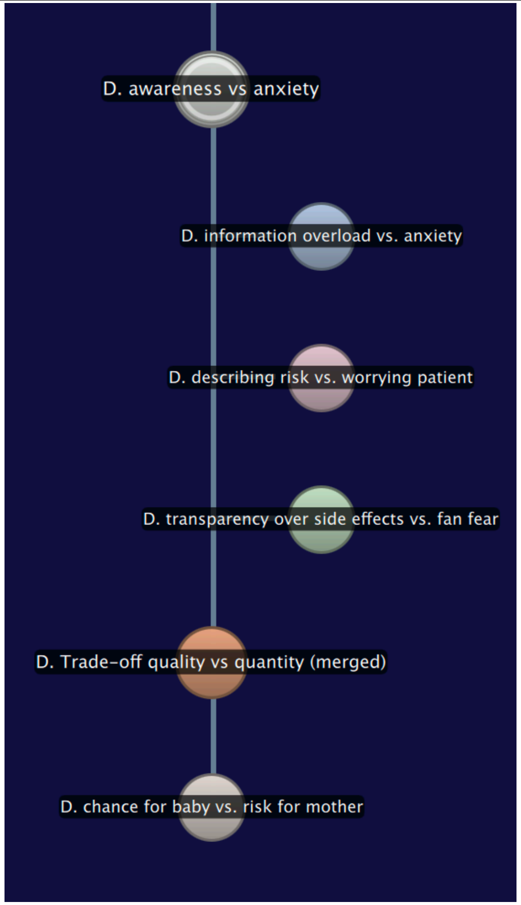 |
| Collection of narratives | During the data analysis process schematic tables were developed, presenting an overview of risk communication strategies with illustrative example sentences (narratives). Risk communication narratives were defined as illustrative example sentences that were used in the consultation room. As the quotes are not anonymized, an insight can only be given in S3 Appendix: Tables Risk communication strategies and narratives. |

<table>
<tr>
<td>Abstraction process</td>
<td>After grouping all codes and reducing them through merging them in higher order categories, the findings were further abstracted into a figure requiring several steps. First the abstraction took place in a handwritten format, trying to visualize risk communication strategies as well as content and context factors and their relation to each other. Subsequently a digital figure was created in several abstraction steps. The clinician goals were first captures under the context factor “clinician”. However, during the iterative course of data analysis it became more and more evident that the goals of clinician play a major role in clinicians risk communication and hence need to be illustrated more clearly and visually in the figure.<br><br>
*Abstraction in form of illustration in a figure: First handwritten attempt*<br>
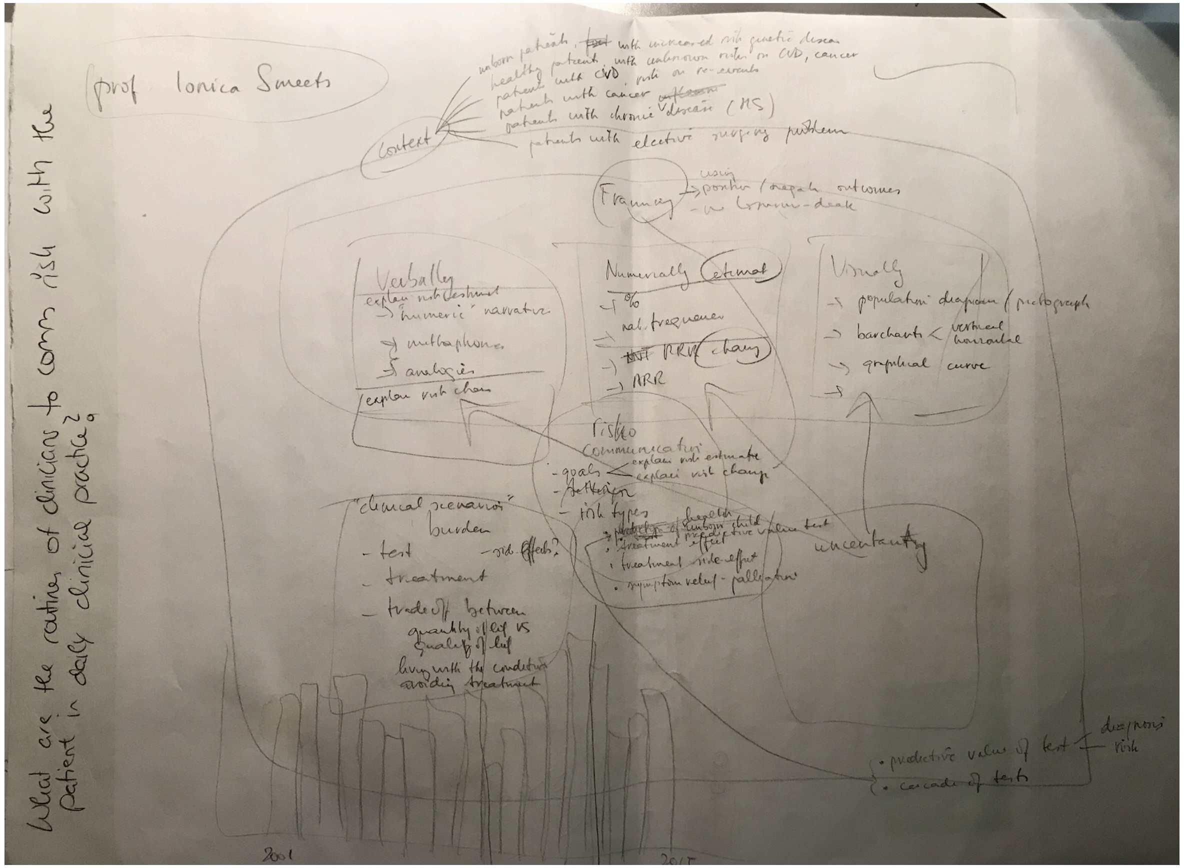

</td>
</tr>
</table>

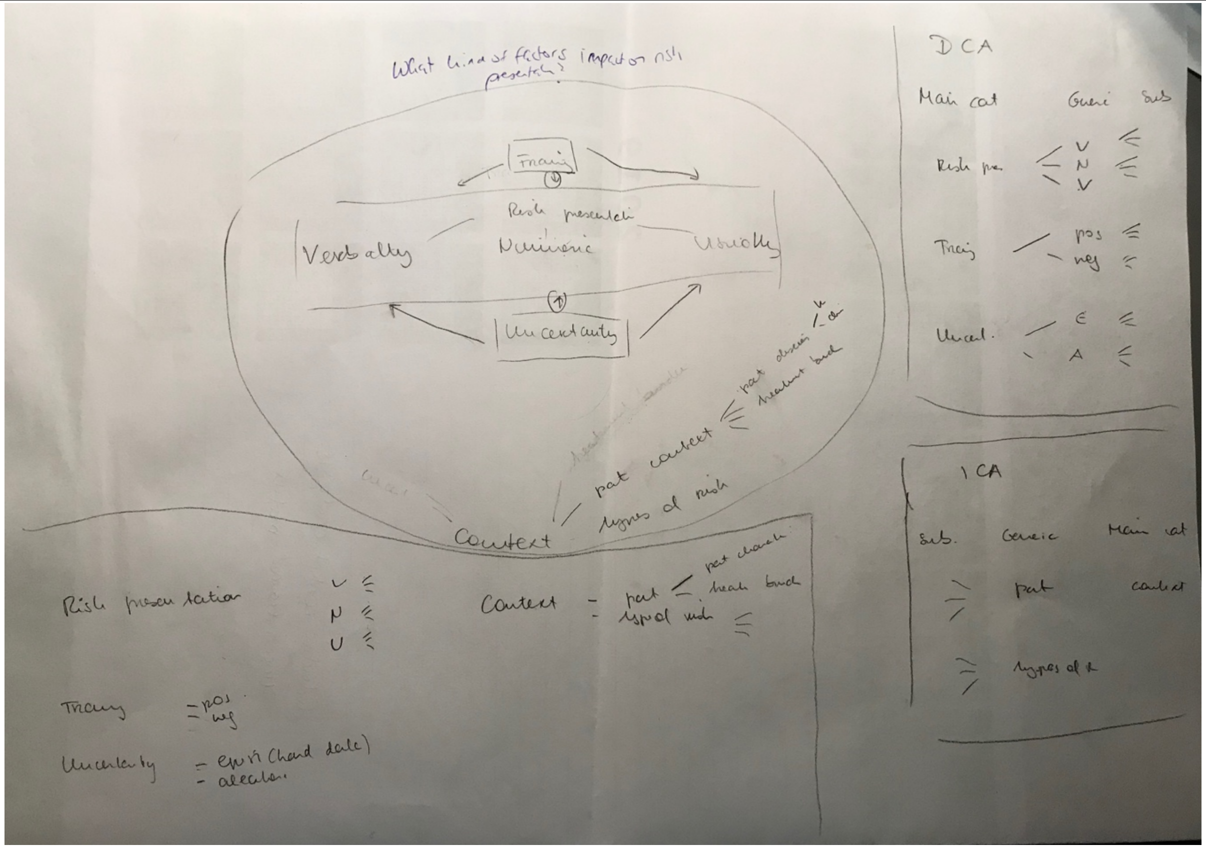
What kind of factors impact on risk presentation?
Risk presentation
Verbally
Numeric
Uncertainty
Context
pat context
DCA
Main cat
ICA
Generic
pat
context
Risk presentation
Context
Uncertainty

| | |
|---|---|
| | *First version of figure incorporating context factors and risk communication strategies*<br>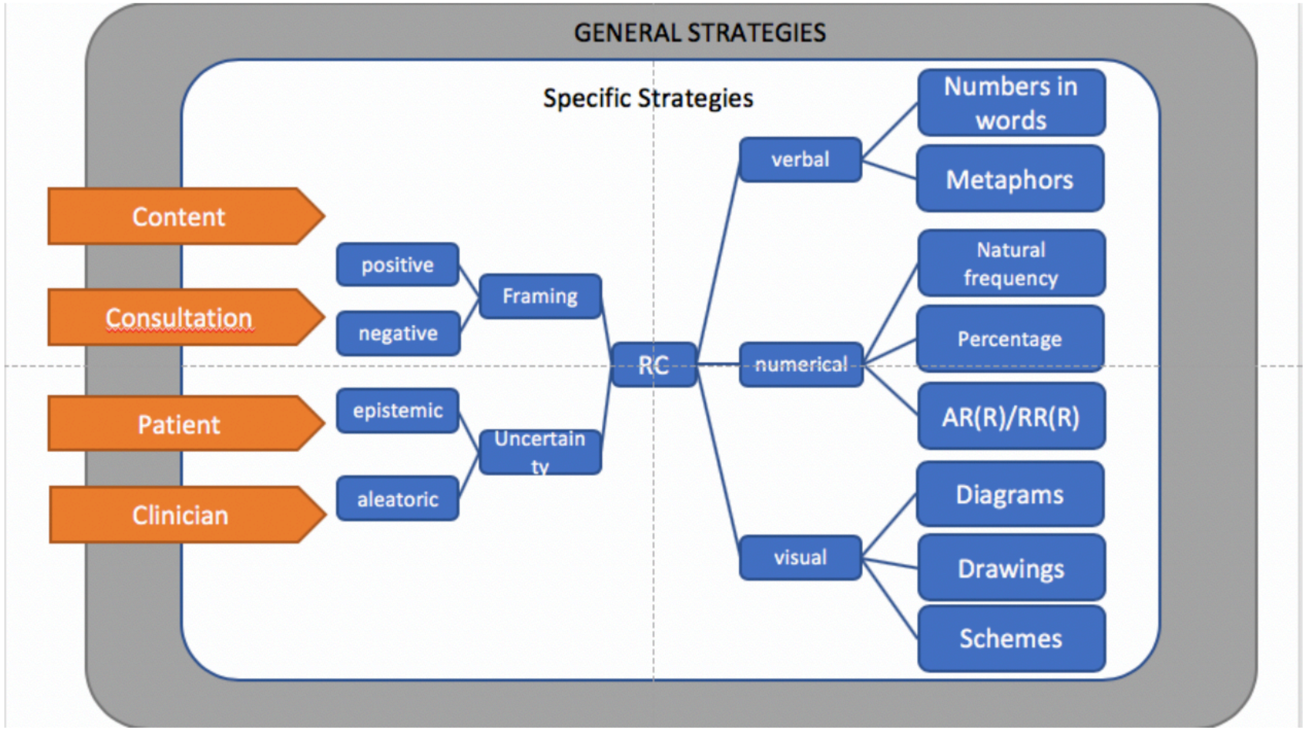<br> |

| | |
|---|---|
| | *Second version of figure incorporating context factors and risk communication strategies*<br>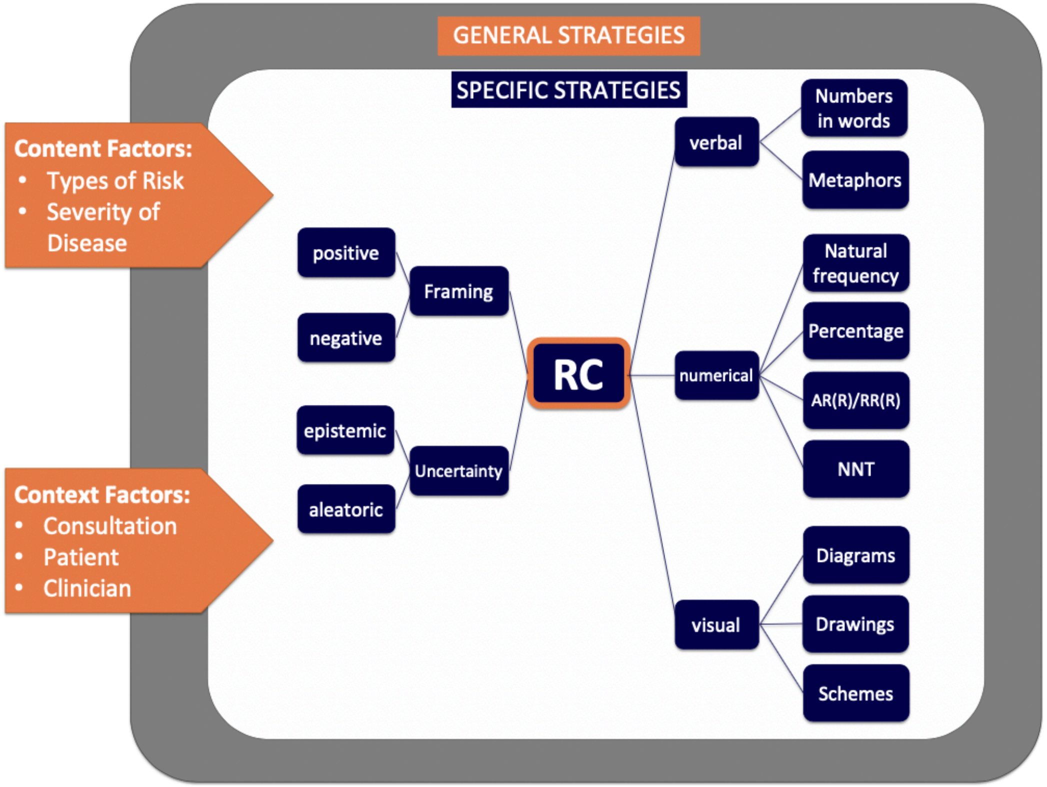<br><br>**Figure 1: Framework Risk Communication (RC)**<br>Blue: deductive part based on literature review; Orange: inductive part emerged during data collection |

| | |
|---|---|
| | *Final Figure showing the interaction of risk communication strategies, communication goals of clinicians and challenges they face in daily clinical practice.* 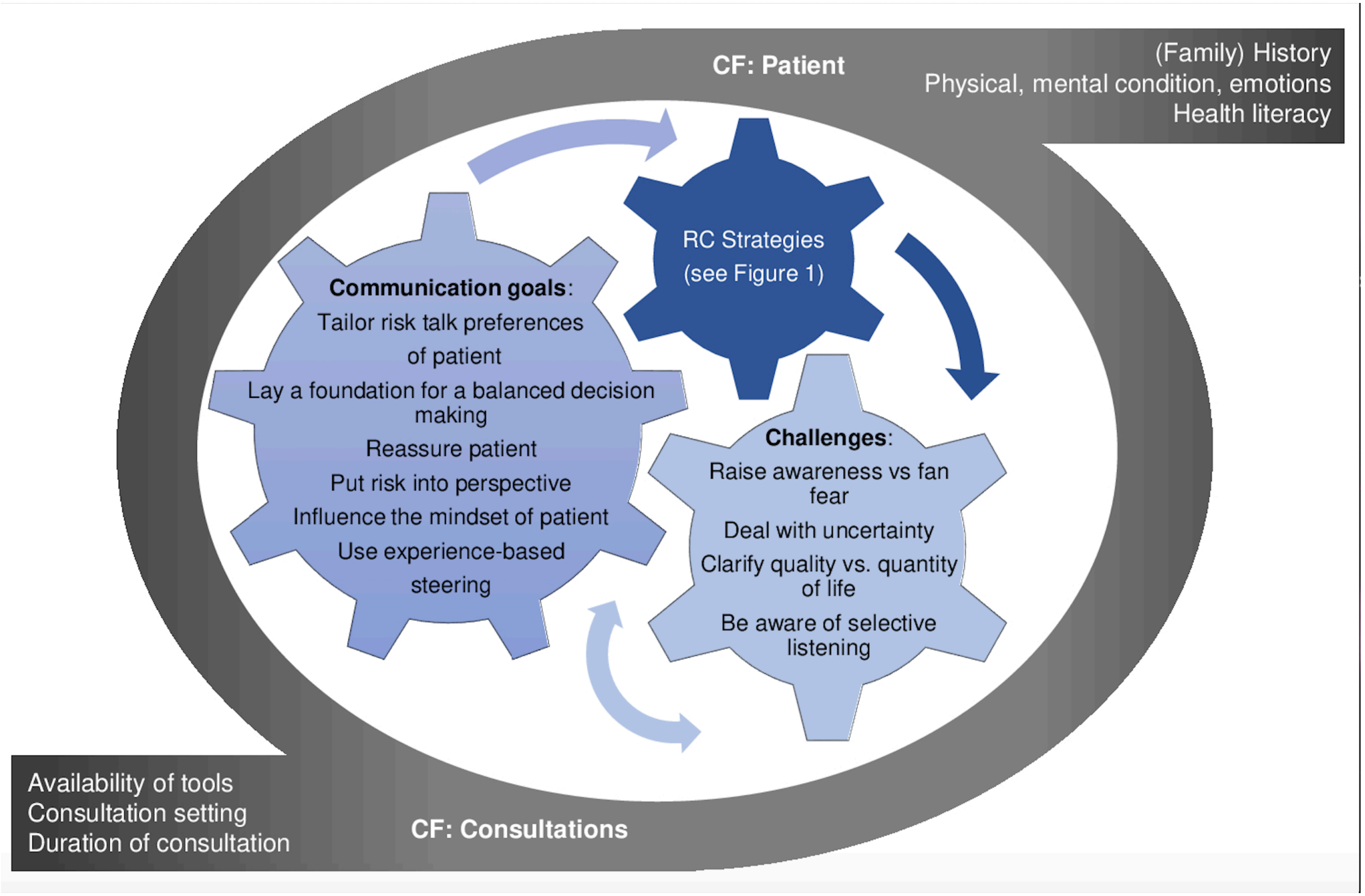  |

## References


Bengtsson M. (2016). How to plan and perform a qualitative study using content analysis. NursingPlus Open 2, 8–14.

Bergman E, de Feijter J, Frambach J, Godefrooij M, et al. (2012). AM Last Page, Academic Medicine, 87(4): 545.

Bradshaw C, Atkinson S, Doody O. (2017). Employing a Qualitative Description Approach in Health Care Research. *Global Qualitative Nursing Research*.

Elo S, Kyngäs H. (2008). The qualitative content analysis process. Journal of Advanced Nursing, 62(1):107–15.

Elo S, Kääriäinen M, Kanste O, Pölkki T, Utriainen K, Kyngäs H. (2014). Qualitative Content Analysis: A Focus on Trustworthiness. *SAGE Open*.

Frambach JM, van der Vleuten CPM, Durning S. (2013). AM Last Page: Quality Criteria in Qualitative and Quantitative Research. Academic Medicine, 88(4):552.

Giroldi E, Veldhuijzen W, Geelen K, Muris J, Bareman F, Bueving H. (2017). Developing skilled doctor–patient communication in the workplace: a qualitative study of the experiences of trainees and clinical supervisors. Advances in Health Sciences Education, 22(5):1263–78.
