# Supplementary material for: A qualitative exploration of clinicians’ strategies to communicate risks to patients in the complex reality of clinical practice
Source: PLoS One. 2020 Aug 13;15(8):e0236751. doi: 10.1371/journal.pone.0236751 (PMC7425874; doi:10.1371/journal.pone.0236751)
Supplement: Attachment — Submitted filename: Response to Reviewers.docx [file pone.0236751.s006.docx]

Andrew Soundy

Academic Editor

PLOS ONE

Dear Dr. Soundy,

Subject: PONE-D-20-06323

A qualitative exploration of clinicians’ strategies to communicate risks to patients in the complex reality of clinical practice

Thank you for your e-mail dated 11 May 2020 enclosing your and the reviewer’s comments. We are very grateful for your valuable feedback and the opportunity to improve our paper through revision. We have carefully reviewed the comments and have revised the manuscript accordingly.

Our responses are given in a point-by-point manner in the enclosed table. In the left columns you will find your and the reviewer’s comments. In the right columns of the table you will find our response and explanation concerning the comment, followed by the changes made in relevant text passages. These changes in the text are highlighted in yellow. The page numbers and lines that correspond to where the change can be found in the manuscript are indicated as well. In the manuscript document the adjustments have been indicated via track changes. An AUDIT trail has been made available in a separate file, named S4_Appendix. The COREQ checklist has been attached in the S5_Appendix. We found a small typing error in Fig 2 and in S3 Appendix, therefore we corrected them and uploaded new versions.

We hope the revised version is now suitable for publication in PLOS ONE and are looking forward to hearing from you.

On behalf of the co-authors,

Yours sincerely,

Romy Richter, Junior Researcher

enclosed:

- Table with reaction to comments of editor and reviewer

# Table with reaction to the comments of editor and reviewer

| **Editor comments to author** | |
| --- | --- |
| Please ensure that your manuscript meets PLOS ONE´s style requirements, including those for file naming. | I adjusted the following things:   - Changed name “Manuscript-RC” to “Manuscript” - Adjusted the headings so that only the first word is capitalized - Table text has been formatted in black - Supplementary information has been mentioned after references - Separate heading for authors contribution in manuscript text has been deleted |
| Please provide additional details regarding participant consent. In the ethics statement in the Methods and online submission information, please ensure that you have specified how verbal consent was documented and witnessed. | We explained the procedure of informed consent more in detail. Under ethical approval and informed consent p.5, line 131-136 and data collection method p.6, line 167-169.  p.5, line 131-136: The NVMO Ethical Review Board granted approval for our study. Clinicians were informed about the study via e-mail. In this e-mail the research was described, and the goal was stated, and the clinicians were asked whether they like to participate in a qualitative interview. Subsequently, clinicians gave written informed consent (via e-mail) whether they were willing to participate. Further, verbal consent was obtained before the start of the interview, just before the audiotape had been started. Verbal informed consent was given for audiotaping and using the data for publication in a scientific journal. Verbatim transcripts of the recorded interviews were anonymized with codes.  p.6, line 167-169: The primary female researcher (RR) carried out semi-structured qualitative interviews (52) at a location preferred by the interviewee. The participants were not known to the primary researcher (RR). Based on the literature review, a semi-structured interview guide (S1 Appendix) with open-ended questions was developed. In the introduction section of the interview participants were again informed about the goal of the research and consequently asked whether they would like to participate (S1 Appendix). Verbal consent was obtained in the interview session just before the audiotape was started. |
| Mention and make sure you adhere to a qualitative framework and mention which one e.g., O’Brien et al., 2014 or Tong et al., 2007 and include in a supplementary file | We clarified this aspect by mentioning the qualitative framework in the manuscript text p.5, line 128-129. We attached the filled-in COREQ checklist as S5 Appendix.  p.5, line 128-129: We conducted a qualitative descriptive study with semi-structured interviews. We took the ontological position of relativism, which holds the view that multiple subjective realties exist. Consequently, we chose the epistemological orientation of subjectivism referring to the existence of many interpretations of reality, and to clinicians’ risk communication strategies being subjectively developed in relation to the clinical situation (48). The consolidated criteria for reporting qualitative research (COREQ) has been used to guide the reporting of the research (49). |
| Identify the ontological position of your selected methodology too please | You have raised an important aspect. We have added the information in a sentence that explicitly mentions the ontological orientation p.5, line 124-125.  p.5, line 124-125: We conducted a qualitative descriptive study with semi-structured interviews. We took the ontological position of relativism, which holds the view that multiple subjective realties exist. Consequently, we chose the epistemological orientation of subjectivism referring to the existence of many interpretations of reality, and to clinicians’ risk communication strategies being subjectively developed in relation to the clinical situation (48). The consolidated criteria for reporting qualitative research (COREQ) has guided reporting of the research (49). |
| Give detail in supplementary file of any changes to semi structured interview after piloting | In the Interview Guide (S1 Appendix) comments were added that indicate the changes made after piloting. The following comments were added:   - After the first interview we adjusted some numbers in the CVD patient case - After the first interviews it became obvious that the patient cases, were not necessary to make the participants talk about their risk communication experiences. Moreover, most interviewees spontaneous reflected on their own cases with regard to this topic. However, the patient cases were left in the interview guide in case of unexpected need but were not actively integrated in the interview - After the first two interviews the interviewee used the following structure concerning the RC methods: After letting the interview freely talk about his risk communication strategies, the interviewee paraphrased the content and if a certain risk communication category (see figure 1) was missing the interviewer asked explicitly for it. However, phrasing of questions were broad enough to leave the interviewee latitude to answer. - After the first two interviews the terms aleatoric and epistemic uncertainty were actively distinct and used when asking about uncertainty |
| Give examples of the analysis within an audit trail - so examples of each stage so the reader could take the data and follow through - or replicate the stages - no need for full interview manuscripts just examples that show clearly the steps | The AUDIT Trial has been added as a supplementary file (S4 Appendix). |
| Make sure the issues of sample size are considered and fit with your pragmatic position | We carefully considered your comment on the sample size. We acknowledge that small sample sizes have limitations. However, we think the sample size fits with our pragmatic position. It is rather a small sample, but we collected data until data saturation was reached and no new aspects emerged. We are aware that the findings of this small sample give only an explorative insight into the clinician’s experiences and the strategies would need to be confirmed in a larger sample. We have elaborated on this issue and expanded the limitations of the sampling method and sample size in the discussion section p.19, line 555-560.  p.19, l. 555-560: As far as we know no other study has given a comprehensive insight into risk communication strategies and narratives that clinicians report to apparently use in practice, in the way that we have done. We provide a comprehensive overview of a sample of clinicians from the Netherlands and Germany. However, as we interviewed a convenience sample of clinicians that are at least familiar with the concept of SDM, these findings might not be generalizable to clinical practice in general, as other doctors might be less inclined to use the presented risk communication strategies. Most of the interviews were conducted in the mother tongue of the participant (Dutch or German) with the exception of six interviews that were conducted in English. Using the mother tongue of the interviewee facilitates the elicitation of authentic narratives. Nevertheless, a drawback might be that the interviewer has only recently learned Dutch. However, discussing the data in the project team with Dutch speaking team members, strengthened the credibility and scientific quality. Another drawback is the small sample size. The findings need to be confirmed in a larger sample size. However, it needs to be considered that qualitative research aims to give a detailed explorative insight rather than confirming specified hypotheses in a representative sample. We are confident that the results reflect adequately the information provided by this sample of clinicians as recruitment and data collection took place until saturation of data was reached. Further, the utilization of interviews to obtain the risk communication strategies relied on the recall of the clinicians. Observations of consultations would probably have resulted in additional information. As observations were not feasible in our study, we believe that using participants’ self-selected patient cases sufficiently enhanced authentic reflections about their risk communication process. In addition, this study cannot make any inferences and statements about the effectivity of the illustrated strategies. Nonetheless, these findings could facilitate specific hypotheses that could be tested in future experimental and systematic studies. For reasons of feasibility the patient perspective could not be explored in this study. |
| Check if member checking and triangulation fits with your paradigmatic position and please reference this for the reader so I can check this. | Since we took the position that multiple subjective realties exist it leads to the fact that also many interpretations of reality exist (relativism, subjectivism). Therefore, it is of value to conduct a member check as it allows the person who provided the information to check whether the researcher has accurately reported and reflected this information. For this reason, we have sent the Interview transcripts to the interviewees and the first version of the article for feedback. These actions strengthen the trustworthiness of findings and may augment the findings since the interviewee can comment on the accuracy of the transcript and reporting of results (Bradshaw 2017, Frambach 2013, Koelsch 2013, Korstjens & Moser 2018, Lincoln & Guba 1985).  Triangulation can concern different aspects of the research process. In our case we refer to “investigator triangulation” which points to the fact that at least two researchers of the research team were involved in the organizational aspects of the study and the data analysis process. Interviews were independently coded by two researchers (RR and TW or RR and EG) and subsequently discussed. The researcher RR and TW held regular meetings in the whole process of examination of the study and RR and EG especially in the process of data analysis. Further, the entire research team had regular meetings during the analysis and reporting phase of this work (RR, EG, JJ, TW). The multidisciplinary character of the research team with different backgrounds in health sciences, medicine and psychology enriched the data analysis process.  We have rewritten this part of the paragraph on p 7, line 198-202 to clarify the messages we attempt to communicate.  p.7, line 198-202: The iterative process of data collection and analysis allowed the researcher to steadily inform data collection by refining the focus in subsequent interviews. Moreover, re-examining the data in the iterative data analysis led to a continuous meaning making. A member check enabled the participants to comment on the interview transcripts and the first version of the article thus strengthening the credibility of data (Bradshaw 2017, Frambach 2013, Koelsch 2013, Korstjens & Moser 2018, Lincoln & Guba 1985). Transcripts were always independently coded by two researchers (RR and EG or RR and TW) and discussed. Analysis and interpretation were additionally discussed in the research team (investigator triangulation) (Frambach 2013, Korstjens & Moser 2018).  References   - Bradshaw, C., Atkinson, S., & Doody, O. (2017). Employing a Qualitative Description Approach in Health Care Research. *Global qualitative nursing research*, *4*, 2333393617742282. https://doi.org/10.1177/2333393617742282 - Frambach, J. M., van der Vleuten, C. P., & Durning, S. J. (2013). AM last page. Quality criteria in qualitative and quantitative research. *Academic medicine: journal of the Association of American Medical Colleges*, *88*(4), 552. https://doi.org/10.1097/ACM.0b013e31828abf7f - Koelsch, L. E. (2013). Reconceptualizing the Member Check Interview. *International Journal of Qualitative Methods*, 168–179. <https://doi.org/10.1177/160940691301200105> - Korstjens, I., & Moser, A. (2018). Series: Practical guidance to qualitative research. Part 4: Trustworthiness and publishing. *The European journal of general practice*, *24*(1), 120–124. https://doi.org/10.1080/13814788.2017.1375092 - Lincoln, Y. S., & Guba, E. A. (1985). Naturalistic inquiry. Beverly Hills, CA: Sage. |
| **Reviewer 1 comments to author** | |
| **Abstract** - Suggest there is a word order issue: ‘but fan fear of anxiety’ should be ‘but fear fanning anxiety’. | Thank you for your suggestion. In the abstract p.2, line 40 it says fan fear **or** anxiety in patients. We referred to fear and anxiety since it does not necessarily always result in fanning fear but possibly a milder form, namely anxiety. In order to prevent confusion, we decided to rephrase the sentence on p.2, line 38-40.  p.2, line 40: Clinicians try to lay a foundation for balanced decision-making and to incorporate patient preferences while faced with several challenges such as the dilemma of raising awareness but triggering anxiety or fan fear in patients. |
| **Background**  Lines 76-77: the choice of ‘manipulation’ is quite strong and implies a negative intention on the behalf of the clinician to my reading. The data (quotes) however from the participants suggest the choice of framing is considered and patient oriented. Alternatively, ‘Choice of framing can influence’ etc. | Thank you for your suggestion. We have exchanged “manipulation by framing influences…” for “choice of framing can influence…”.  p.3, line 75: Choice of framing can influence the perception of risks and hence the decision made during a clinical consultation (15). |
| **Background**  Communicating risk to patients in daily clinical practice (lines 93-101, p4): most of the references in this section are from general practice, so it was unclear to me whether ‘daily clinical practice’ referred to primary care or was intended to be more generic. However, a major finding was the importance of clinical context (the disease setting as well as patient characteristics), yet this isn’t foreshadowed in this introductory section. Perhaps continue to build the research gap after line 97 by giving examples of the likely risk communication settings/ challenges for different clinical contexts, in which the ‘daily clinical practice’ will look very different. | Thank you for raising this point. ‘Daily clinical practice’ referred to primary and secondary health care. We have enriched the text to reflect more on the importance of the clinical context for our research question in different medical disciplines, p.4, line 91-104.  p.4, line 91-104: Despite theoretical insights into recommendations for preferred risk communication formats (10,12,21,34,41,42), risk communication remains a major challenge (9,12,21,23). The various medical disciplines with their different contexts demand clinicians to nuance the risk talk sensitively to specific aspects of their discipline. For example, in clinical genetics risk communication needs to address predictive testing such as the small chance of getting a false positive test result or the small chance of miscarriage in invasive testing (43). Thus clinical geneticists often have to deal with communicating small and difficult to imagine risks that could have a great impact in the future of the patient. General practitioners often counsel the patient for more general problems in the present, while doctor and patient usually have a closer and continuous relationship with repetitive opportunities for a dialogue and reassurance of the patient. Whereas surgeons often have to communicate the side effects and consequences of severe surgical procedures under time pressure while having seen the patient one or two times in the hospital. On the other hand, oncologists often have to consider the communication of overtreatment and overdiagnosis when discussing screening and treatment options, especially when dealing with frail elderly patients. Undoubtedly, risk communication is a core skill for clinical counsellors in various medical disciplines. However, as far as we know, the literature mainly provides formalistic or standardized language on written communication of numerical and visual risk communication formats, and lacks practical and illustrative examples of how to communicate risks to patients in daily clinical practice in the various contexts. Research emphasizes that trainees are in need of concrete illustrations of meaningful language and graphical examples in order to acquire complex communication skills (44,45). The provision of illustrative risk communication strategies in the SDM concept could support educational training programs. Altogether, this points to a potential gap between theoretical recommendations in literature and ease of application in clinical practice. |
| **Background**  Lines 103 104 p.4, read like the first line of the conclusion. Instead – the aim is to … | Thank you for your suggestion. We agree with you and have adjusted the sentence p.4, line 112-113.  p.4, line 112: The aim is to add to the existing literature on risk communication by gaining a deeper understanding of the clinician’s actual strategies in communicating risk in daily clinical practice. |
| **Background**  I suggest the phrase ‘young doctors’ is replaced by junior doctors or simply ‘trainees.’ They are not always ‘young’ by the time they finish their training! | We agree with you and have exchanged the term “young doctors” for “trainees” in the whole manuscript. |
| **Methods**  Data analysis: I would like some clarification on the inductive analysis. Lines 163, page 6 mentions themes but the reference, 50, is to qualitative content analysis rather than thematic analysis. I suggest the analysis was content analysis. | We indeed used a content analysis. The term “themes” was used as a general term and did not refer to the thematic analysis. However, we agree that using the term “themes” in relation with content analysis leads to confusion. In order to prevent this, the word “themes” has been exchanged through the word “aspects“, in the sentence p.6, line 180-182.  p.6, line 181: All interviews were audiotaped and transcribed verbatim. Additionally, drawings made by interviewees were added to the transcript. We started with a deductive content analysis to gather risk communication strategies according to the categories described in the framework. Collaterally, the inductive approach was used to capture and group new aspects concerning clinician goals, challenges and dilemmas, risk communication strategies and content and context-related factors with no preconceived ideas (53). |
| **Methods**  The section on techniques to enhance trustworthiness is exemplary because it covers a range of processes in the methods, not just analysis. For the analysis, however, the data analysis sentence p. 177 refers to going back to the interviews. There is little on how the transcripts were coded for the inductive component -here and in the preceding data analysis section. This could be elaborated. | To clarify the process of data analysis we have added a supplementary file (S4 Appendix) with an AUDIT Trail to explain the analysis process in more detail. |
| **Results**  Pl0, line 238. Should it read So […] to too quickly going through.. evaluative too, or to? Both work but different meanings. | We double-checked the quote. The main message is “going too fast through the circle of SDM”, in order to make it clearer we adjusted the word order of the quote, p.10, line 257-261.  p.10, line 258: We are actually moving pretty quickly from risk communication to “*Well, these are your options - and what are we going to do?”*. So [...] going too quickly through the circle of shared decision-making. While [...] if people understand this [risk communication] well, I think they are more receptive to the following steps [of shared decision making].  -- Interview C04 General Practice -- |
| **Results**  The finding in line 260 p. 11 is important and is supported by the quotes; I don’t think this findings is sufficiently reflected in the abstract ie noted the ‘goal oriented’ nature of the clinician’s communication is really part of the SDM (informed by the patient’s goal) and not a paternalistic, clinician centred goal. | You have raised a core aspect of our findings that apparently needs more clarification. Our findings show that next to the risk communication strategies, clinicians also use specific communication goals. The goal-directed nature of risk communication refers to those specific communication goals that are applied in the risk communication process next to the risk communication strategies. One specific communication goal is indeed “patient-driven” as the clinicians often try to tailor the risk talk to the preferences of the patient. However, those specific communication goals do not necessarily have to be “patient preference-driven”. Some goals also depend on the clinician’s preferences and expertise e.g. “use of experienced based steering through the clinician” can be applied to influence the lifestyle of the patient or to make high risk patients thoroughly consider the side effects of a treatment. We have revised the paragraph about the goal-directed and context-specific nature in the discussion. Please also see the next row of this table.  In the abstract we added the goal “tailor the risk talk to the preferences of the patient” in the sentence p.2, line 38-40.  p.2, line 39: However, it became clear that clinicians’ expertise and communication goals also play an important role in the risk talk. Clinicians try to lay a foundation for balanced decision-making and to incorporate patient preferences while faced with several challenges such as the dilemma of raising awareness but triggering anxiety or fan fear in patients. |
| **Discussion**  P. 19 for clarification: in the recommendation for practice “therefore, an holistic, context-specific and goal-directed approach…” do the authors mean that the approach to risk communication should be sensitive to context rather than context specific, where context specific refers to teaching risk communication approaches tailored to a particular clinical discipline? I think the message is the former rather than the latter as it is the principle. It might also be worth clarifying here and in the conclusion (line 546) that the clinician’s communication goals are informed by the goals of the patient. | Thank you for your input. As you already assumed, “context-specific” refers to the fact that risk communication should be sensitive to the context. In order to clarify this matter of phrasing, we added an explanation of our definition of context-specific, p.18, line 509-517.  p.18, line 509-517: Previous literature on doctor-patient communication empirically supports both the context-specific and goal-directed nature of communication (67-71). As presented in this study, the risk communication process is influenced by different contextual factors at the consultation, patient and clinician level. Risk communication has to be sensitive to the context of the consultation (context-specific), e.g. concerning practical aspects such as time pressure or patient aspects such as their emotions. Risk communication is also goal-directed as clinicians apply specific communication goals in the process. Those specific communication goals vary and might depend on e.g. patient preferences, but they can also be driven by clinicians’ preferences and expertise. For example, clinicians might aim to influence the lifestyle of the patient or to make high risk patients thoroughly consider the side effects of a treatment. Clinicians adapt how they present risk accordingly. Therefore, risk communication is not merely to be satisfied with a standardized stepwise approach of the “right” risk communication format/strategy, considering the uniqueness of situations in daily clinical practice. A holistic, context-specific and goal-directed approach to teaching risk communication seems to be needed. |
